# Neoadjuvant chemotherapy versus primary surgery in advanced ovarian carcinoma

**DOI:** 10.1186/1477-7819-3-57

**Published:** 2005-08-31

**Authors:** Mohamed AF Hegazy, Refaat AF Hegazi, Mohamed A Elshafei, Ahmed E Setit, Maged R Elshamy, Mohamed Eltatoongy, Amal AF Halim

**Affiliations:** 1 Surgical Oncology department, Mansoura University, Mansoura, Egypt; 2 Obstetrics and Gynecology department, Mansoura University, Mansoura, Egypt; 3 Department of Medicine, University of Pittsburgh, Pittsburgh, PA, USA

## Abstract

**Background:**

Patients with advanced ovarian cancer should be treated by radical debulking surgery aiming at complete tumor resection. Unfortunately about 70% of the patients present with advanced disease, when optimal debulking can not be obtained, and therefore these patients gain little benefit from surgery. Neoadjuvant chemotherapy (NACT) has been proposed as a novel therapeutic approach in such cases. In this study, we report our results with primary surgery or neoadjuvant chemotherapy as treatment modalities in the specific indication of operable patients with advanced ovarian carcinoma (no medical contraindication to debulking surgery).

**Patients and methods:**

A total of 59 patients with stage III or IV epithelial ovarian carcinomas were evaluated between 1998 and 2003. All patients were submitted to surgical exploration aiming to evaluate tumor resectability. Neoadjuvant chemotherapy was given (in 27 patients) where optimal cytoreduction was not feasible. Conversely primary debulking surgery was performed when we considered that optimal cytoreduction could be achieved by the standard surgery (32 patients).

**Results:**

Optimal cytoreduction was higher in the NACT group (72.2%) than the conventional group (62.4%), though not statistically significant (P = 0.5). More important was the finding that parameters of surgical aggressiveness (blood loss rates, ICU stay and total hospital stay) were significantly lower in NACT group than the conventional group. The median overall survival time was 28 months in the conventional group and 25 months in NACT group with a P value of 0.5. The median disease free survival was 19 months in the conventional group and 21 months in NACT group (P = 0.4). In multivariate analysis, the pathologic type and degree of debulking were found to affect the disease free survival significantly. Overall survival was not affected by any of the study parameters.

**Conclusion:**

Primary chemotherapy followed by interval debulking surgery in select group of patients doesn't appear to worsen the prognosis, but it permits a less aggressive surgery to be performed.

## Background

The diagnosis and management of ovarian cancer is one of the greatest challenges in oncology. Approximately, half of ovarian carcinoma patients die from the disease making it the leading cause of gynecologic cancer-related death in most industrialized countries [1].

Although our approach and knowledge of epithelial ovarian cancer has changed in the past 25 years, the overall survival has not been affected as approximately 65% to70% of all cases continue to be diagnosed with stage III or stage IV disease. Surgical reduction of tumor bulk has become the preferred first step in the management of advanced epithelial ovarian cancer [2]. Observations that the excision of large tumor masses could provide palliation and a modest extension of life have been recorded for more than 50 years. Enhancement of sensitivity to chemotherapy, yet unproven, could be the greatest benefit of tumor debulking [3].

Approximately 70% of patients present with advanced ovarian cancer, when optimal debulking can not be obtained, and therefore gain little benefit from surgery [4]. On the other hand, patients who are severely compromised medically carry an unwarranted risk to surgery. Neoadjuvant chemotherapy (NACT) has been proposed as a novel therapeutic approach to a variety of solid tumors when the disease is not amenable to surgical resection at the time of diagnosis or the patient is unfit for aggressive debulking surgery [5]. NACT has now been recognized as a useful modality for the treatment of various advanced cancers [6,7]. In cases with advanced ovarian carcinomas, platinum based chemotherapy regimens have been found to produce higher response rates and in some studies have produced a statistically significant survival advantages compared with drug regimens without platinum [8,9].

Thus the two treatment options available for treating patients with advanced ovarian tumor are either a primary surgical cytoreduction or to start with chemotherapy hoping for down staging the tumor and then go ahead with surgery.

In this study, we report our results with these two treatment modalities applied only to operable patients without medical contraindications to surgery. We assessed the patients for different variables, such as the ability to perform optimal debulking, rate of non-standard surgery (Excision of more than one organ), disease free survival and overall survival.

## Patients and methods

This prospective trial included a total of 59 patients with stage III or IV epithelial ovarian carcinomas that were evaluated between 1998 and 2003. Patients who were selected for our study had advanced ovarian carcinoma and were free from severe concomitant medical illness that could preclude surgical interference (such as those with WHO performance status 2, or 3). Patient characteristics are summarized in Table 1. Patients were subjected to physical examination, serum level of CA 125 measurement, radiological studies, and histopathological confirmation of ovarian carcinoma. All patients were submitted to surgical exploration at the Surgical Oncology Unit, Mansoura University Hospitals. The purpose of this exploration was to evaluate tumor resectability; to perform primary debulking surgery when optimal cytoreduction seemed feasible and to treat primary unresectable tumors with neoadjuvant chemotherapy. Optimal debulking has been variously defined, however we adopted the Gynecologic Oncology Group definition which defines it as leaving residual disease of less than 1 cm [10]. This strategy was explained to the patients and informed consents were obtained. Surgical exploration was usually done laparoscopically (38 cases) unless it was contraindicated, when laparotomy was done (21 cases). Neoadjuvant chemotherapy was given (in 27 patients) when we considered that optimal cytoreduction was not feasible with the standard surgery, defined as 1) total abdominal hysterectomy with bilateral salpingoophorectomy, 2) appendectomy, 3) total infragastric omentectomy, 4) peritonectomy limited to the pelvis, paracolic gutters, anterolateral diaphragmatic area, and 5) pelvic, common iliac, and infrarenal paraaortic lymphadenectomy. Conversely primary debulking surgery was performed when we considered that optimal cytoreduction could be achieved by the standard surgery (32 patients). However, in a few cases non-standard surgery, meaning a single organ resection (e.g., small intestine, colon, spleen) in the way to achieve an optimal cytoreduction was adopted. All patients who underwent intestinal surgery were evaluated by a single surgeon (MH).

**Table 1:** Patient characteristics

	**Conventional(n = 32)**	**NACT(n = 27)**	**p**
**Age**	53.6 ± 9.8	58.7 ± 4.6	NS
**FIGO stage**			
***IIIc***	14 (43.8%)	11 (40.7%)	NS
***IV***	18 (56.2%)	16 (59.3%)	NS
**Grade**			
***II***	14 (43.8%)	12 (44.4%)	NS
***III***	18 (56.2%)	15 (55.6%)	NS
**Histological type:**			
***Serous***	9 (28.1%)	7 (25.9%)	NS
***Mucinous***	13 (40.6%)	10 (37%)	NS
***Undifferentiated***	10 (31.3%)	10 (37%)	NS
**Staging procedure**			
***Laparoscopy***	21 (65.6%)	17 (62.9%)	NS
***Laparotomy***	11 (34.4%)	10 (37.1%)	NS

Chemotherapy regimens were all platinum based and included cisplatinum 75 mg\m^2 ^plus cyclophosphamide 600 mg\m^2 ^(repeated every 3 weeks). This regimen was applied to all cases of the NACT group and was applied in all cases of the conventional group postoperatively.

The response to neoadjuvant chemotherapy was evaluated after the third cycle. Clinical response to chemotherapy was evaluated on clinical examination, serum CA 125 level, and computed tomography (CT) scan. Tumor response was classified according to the WHO criteria. [11]. Patients were then referred for second surgical cytoreduction when they presented no signs of progression during chemotherapy (18 cases). The surgicopathologic response to NACT was assessed at secondary surgery. Nine patients progressed under chemotherapy and were not surgically operated. The aggressiveness of surgical cytoreduction was evaluated in terms of the blood loss rates, and the length of intensive care unit and postoperative hospital stay. A report of the peroioperative and postoperative complications in both groups was recorded.

Survival curves since diagnosis (first surgical procedure) were calculated according to the Kaplan Meier method, and survival curves were compared by the log-rank test.

## Results

**Figure 1:**
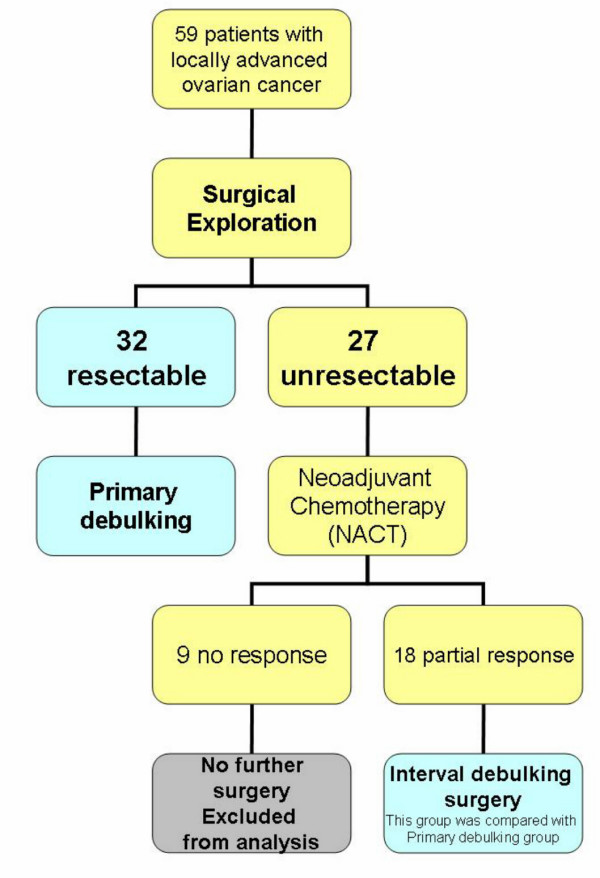
Treatment plan according to the results of initial exploration.

Between April 1998 to January 2003, 59 patients presented with operable, locally advanced epithelial ovarian carcinoma. After surgical exploration, 32 patients seemed resectable and primary cytoreductive surgery was carried out, and 27 patients seemed unresectable and neoadjuvant chemotherapy was given to them. Among those patients, 9 progressed during chemotherapy and were not operated (Figure 1)

Patient characteristics are summarized in Table 1. When comparing the NACT patients with the conventionally treated patients as a group, the NACT group were statistically older (58.7 ± 4.6 years vs. 53.6 ± 4.6 years) than the conventional group (P = 0.04)

The staging procedure was laparoscopy in 38 patients and laparotomy in 21 patients. In the NACT group (n = 27), the mean interval between surgical staging and the start of chemotherapy was 13.7 days (range 5–33) days after laparoscopy and 18.9 days (range 7–41 days) after laparotomy (p = 0.055).

All patients then received platinum-based chemotherapy. The median number of neoadjuvant chemotherapy cycles was 3 (range 2–6). Only 2 patients showed partial response after 2 cycles and cytoreductive surgery was done. Twenty-five patients received 3 or more cycles. Among 27 patients of this series, 18 (66.6%) responded to NACT according to clinical examination, serum CA 125 level and abdominopelvic CT scan. All of them showed partial response. Conversely 9 patients showed no response (4 cases showed a stable disease and 5 cases showed progressive disease). Those 9 cases were not operated and referred to continue chemotherapy.

We aimed to compare the cases who were primarily operated (n = 32) and those who were operated after responding to neoadjuvant chemotherapy (n = 18) in terms of the degree of optimal debulking and the morbidity associated with surgical procedures.

In NACT group, optimal cytoreduction was achieved in 13 cases. Thus the optimal debulking rate was 48.1% among the overall number of patients in this group (n = 27) and 72.2% among those who were operated (n = 18). In the conventional group the optimal debulking rate was (62.4%). The difference was statistically insignificant (P = 0.5). However, one must remember that 9 patients progressed while on chemotherapy and were not operated (Table 2).

**Table 2:** Degree of debulking in the conventional group and the surgically operated patients in NACT group

	**Conventional Group (n = 32)**	**NACT (n = 18)**
Optimum cytoreduction	20(62.5%)	13(72.2%)
Suboptimal cytoreduction	12(37.5%0	5(27.8%)

In the conventional group, non-standard surgery was performed in 11 cases (34.4%), and in 4 cases (27.8%) of NACT group (Table 3). Resection and primary anastomosis of the small intestine occurred in 10 patients, partial cystectomy was done in 3 cases, colectomy was done in 4 cases, and splenectomy in 2 cases. Multiple organ resections (MOR) occurred in two cases in the conventional group and in one case in NACT group.

**Table 3:** The frequency of non-standard surgeries in both groups

	**Conventional Group (n = 32)**	**NACT Group (n = 18)**	**p**
**Number of patients**	11 (34.4%)	4 (22.2%)	NS
**Organs resected**			
***Small intestine***	7	3	
***Colon***	3	0	
***Bladder***	2	1	
***Spleen***	1	1	

Patients in NACT group showed a significantly less blood loss rates (p = 0.02), less ICU stay (p = 0.03), and less total hospital stay (p = 0.05). There was no difference between perioperative morbidity and mortality in the two patient groups (Table 4). Complications were more in the cases that underwent intestinal surgery (3 cases of wound infection, 6 cases of fever, and 3 cases of DVT).

**Figure 2:**
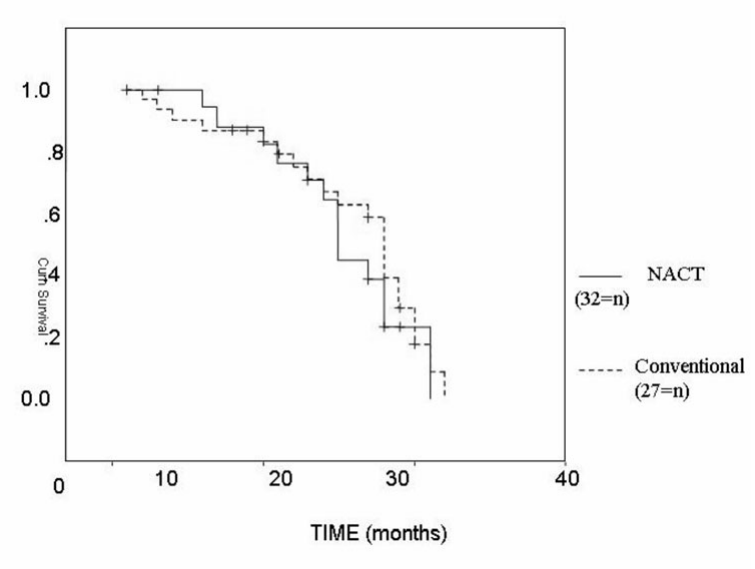
Overall survival in NACT and conventional groups.

**Figure 3:**
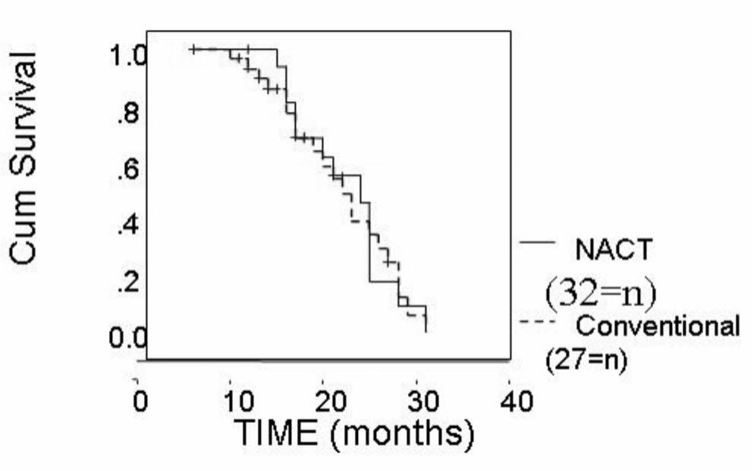
Disease free survival in NACT and conventional groups.

On assessment of the survival we compared the whole number of both groups i.e., we added the 9 patients who were not surgically operated in NACT group. The median overall survival time was 28 months in the conventional group and 25 month in the NACT group with an insignificant P value (P = 0.5) (Figure 2). The median disease free survival was 19 months in the conventional group and 22 months in the NACT group with an insignificant P value (P = 0.4) (Figure 3)

**Table 4:** Parameters of surgical morbidity in both groups

	***Conventional Group (n = 32)***	***NACT Group (n = 18)***	***p value***
**Duration of surgery**			
***Mean***	190	150	NS
***range***	70–350	90–270	
**Blood loss rates (cc)**			
***Mean***	735	420	0.02
***Range***	50–5000	50–3000	
**ICU stay (days)**			
***Mean***	4.4	1.7	0.03
***Range***	1–9	1–5	
**Hospital stay (days)**			
***Mean***	15.9	10.5	0.05
***Range***	6–49	4–31	
**Wound infection**	2	2	NS
**Fever > 38.5°C > 3 d**	7	1	NS
**Atelectasis**	1	1	NS
**Pleural effusion**	2	0	NS
**DVT**	3	1	NS

**Figure 4:**
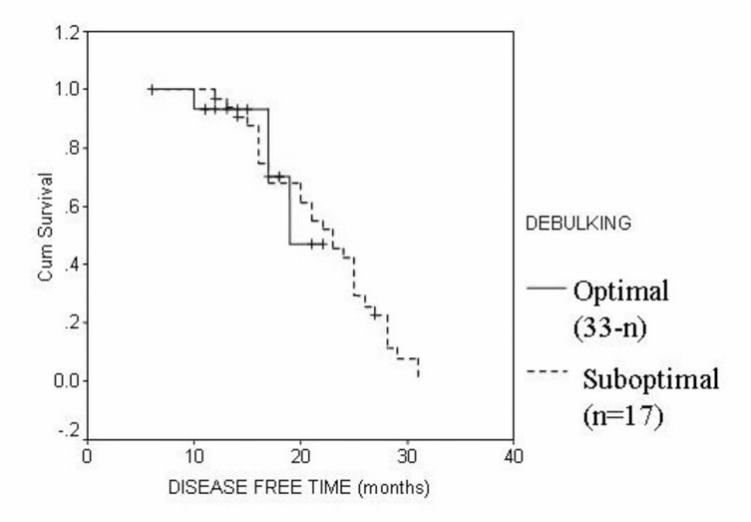
Disease free survival in both groups according to the degree of debulking.

In multivariate analysis of both the conventional group and NACT group, the overall survival was not significantly affected by any of the study parameters (pathologic type, grade, stage, degree of optimal debulking). In the conventional group, the disease free survival was significantly affected with the degree of optimal cytoreduction only (P = 0.001) (Figure 4). In the NACT group the disease free survival was significantly affected by the tumor type (P = 0.02) and the degree of optimal debulking (P = 0.01).

## Discussion

The clinical basis of aggressive cytoreductive surgery in the initial management of ovarian cancer is the significantly improved survival gained by those patients in whom optimal cytoreductive surgery was accomplished [12,13]. The presence of residual disease after surgery is one of the most adverse prognostic factors for survival. Therefore, although the definition of optimal cytoreduction has been modified over the last two decades, it is generally agreed that every attempt should be made to surgically resect as much disease as safely possible [4].

The value of debulking after induction chemotherapy has been largely debated in the last decades. Recently several investigators introduced the concept of interval debulking surgery meaning a surgical procedure with debulking intent foreword and followed by cytoreductive chemotherapy [14,15]. Based on the GOG 152 data, interval debulking surgery does not seem to be indicated in patients who underwent primarily a maximal surgical effort by a gynecological oncologist [15].

In a population of patients with advanced ovarian carcinoma who deemed unresectable by surgical exploration, neoadjuvant chemotherapy helped to select patients for feasible and relatively less aggressive interval debulking. Patients who did not respond or progressed under chemotherapy were spared surgery [16].

An issue of importance is which criteria should be used to define the respectability of the tumor and consequently the selection of which patients might benefit from NACT approach. Imaging (computed tomography scan) based criteria have been developed by different authors [17,18]. Nelson *et al *[17] showed that the predictive value of a positive test (CT scan demonstrating non respectability) was only 67%. Bristow et al developed a predictive index that was able to correctly predict surgical outcome (optimal < 1 cm versus suboptimal residual disease status) [19]. The specificity or the ability to identify patients undergoing optimal debulking was 80%. The authors agree with Ansquer *et al *[8] and Vergote *et al *[14] in that a laparoscopy and in certain situations exploratory laparotomy provides certain advantages as a selection tool. It allows for making a histological diagnosis and objectively documents the extent of the disease. At the same time it identifies patients who can be optimally debulked, thus not denying the possible benefit of such a procedure. The issue of port site implantation in this patient group can probably be addressed by proper technique (Closure of the peritoneum and excision of trocar port site at the definitive surgery) and immediate (< 1 week) start of chemotherapy [19]. In this study we performed laparoscopy as a selection tool in most patients unless it was contraindicated when a laparotomy was done. The limits of standard debulking surgery that were found at exploration were extensive bowel involvement, large involvement of the peritoneum located in the upper abdomen particularly in the dorsal diaphragmatic area, and liver metastasis. These cases were referred for neoadjuvant chemotherapy. Initiation of chemotherapy was significantly delayed in the laparotomy group than the laparoscopy group. No case presented with port site recurrence in the laparoscopy patients.

Chemotherapy was platinum based. The number of preoperative cycles ranged from 2–6. Most patients were explored after 3 cycles. It is noteworthy to mention that three patients who were explored after 5–6 cycles tended more frequently to present no microscopic disease. Indeed the optimal number of chemotherapy cycles to be given before planned surgery is still a major, unresolved issue. In previous published studies the number of preoperative chemotherapy cycles ranged from 2–10 [20-22]. It seems that the chance of achieving an optimal debulking increases in responding patients with the numbers of cycles before surgery [23]. This potential advantage has to be balanced against the risk of emergence/ selection of drug resistant cell clones and cumulative drug toxicity associated with the increased number of chemotherapy cycles [20].

Eighteen patients of NACT group had a clinical response. Optimal cytoreduction rate was 48.1% among the overall number of patients in this group (n = 27) and 72.2% among those who were operated (n = 18). This correlates with previous reports of Jacob *et al *[12] who reported optimal cytoreduction in 77 % of patients and Surwit *et al *[24] who reported 55% of cytoreduction less than 1 cm.

We believe that the benefit of neoadjuvant chemotherapy does not lie in its ability to obtain larger percentage of optimal cytoreduction because the increased and the more widespread use of newer technologies as ultrasonic aspirator, argon beam coagulator and ultra radical surgical procedures could increase the fraction of patients who are optimally debulked upfront but at the likely cost of increase morbidity [24].

The value of neoadjuvant chemotherapy is to obtain optimum cytoreduction by means of less aggressive surgery. In our study, debulking surgery in NACT group was less aggressive than in the conventional group with less blood loss rates, shorter intensive care stay and shorter postoperative hospitalization. These finding are consistent with the data of Schwartz *et al *[23] who reported that the aggressiveness of debulking surgery seems to be decreased after neoadjuvant chemotherapy. There was no significant difference between perioperative morbidity and mortality in the two patient groups.

In our study, the median overall survival time yields no significant difference in both groups. Onnis *et al *[25] described 88 patients treated with NACT compared with 248 patients treated with upfront surgery followed by chemotherapy. The overall survival was not improved. In an analysis by Surwit *et al*, the median survival of 29 patients who underwent primary chemotherapy was 22 months, which the author said was similar to that of patients who undergo primary surgery [24]. Schwartz *et al *[21] reported on 59 patients treated with NACT of whom 41 were eventually operated compared with a control group of conventionally treated patients, the patients receiving NACT were significantly older and had a poor performance status but still obtained a similar survival. Vergote *et al *[14] reported that NACT resulted in survival rates in selected patients with advanced ovarian cancer that were comparable to those associated with primary cytoreductive surgery.

Conversely, Kuhn *et al *[26], Rose *et al *(GOG 152) [15], and Muggia *et al *(GOG 158) [27] reported prolonged survival times and significantly better median survival in NACT group than the conventionally treated patients. This controversy might be attributed to different patient characteristics and different treatment modalities.

In our study, there was no significant difference in the median disease free survival between both groups. Our results are similar to those of Kayikcioglu *et al *[28] who reported a disease free survival of 16.03 (0–84 months, median= 12 months) in 158 patients with advanced ovarian carcinoma treated by conventional surgery. In 145 patients who received NACT, he reported a disease free survival of 13.9 ± 10.12 months (0–48 median 13.9 months).

## Conclusion

Primary cytoreductive surgery is still the gold standard in the treatment of ovarian carcinoma [29]. Neoadjuvant chemotherapy for advanced unresectable ovarian carcinoma lead to the selection of a group of patients sensitive to chemotherapy, in whom secondary cytoreductive surgery can be achieved in a less aggressive manner. Also neoadjuvant chemotherapy prevents mutilating surgery in patient with a very poor prognosis either because of progressive disease or because of primary chemoresisetence. These findings must be confirmed by a larger prospective study. A large randomized trial evaluating the efficacy and morbidity of primary surgery versus neoadjuvant chemotherapy followed by interval debulking surgery is ongoing.

## Competing interests

The author(s) declare that they have no competing interests.

## Authors' contributions

**MAFH: **obtained full data about the patients, applied the study design, performed surgical interference to the patients, searched literature and drafted the manuscript,

**RAFH: **shared in the study design, performed statistical work and helped to draft the manuscript and edited the final version.

**MAE: **obtained patient consent and shared in surgical interference to the patients.

**AES,: **shared in surgical interference to the patients and in collecting their data.

**MRE**: shared in surgical interference to the patients and in collecting their data

**ME **shared in surgical interference to the patients and in collecting their data,

**AAFH,: **gave the patients chemotherapy protocol and followed them up.

## Funding

This work was supported by an internal funding from the Oncology Center, Mansoura University, Mansoura, Egypt.
